# Presence of a Novel Anatomical Structure May Cause Bleeding When Using the Calyx Access in Mini-Percutaneous Nephrolithotomy

**DOI:** 10.3389/fsurg.2022.942147

**Published:** 2022-06-21

**Authors:** Fangyou Lin, Bojun Li, Ting Rao, Yuan Ruan, Weimin Yu, Fan Cheng, Stéphane Larré

**Affiliations:** ^1^ Department of Urology, Renmin Hospital of Wuhan University, Wuhan, China; ^2^ Department of Urology, Robert Debré Teaching Hospital, University of Reims, Reims, France

**Keywords:** renal stones, kidney anatomy, calyx access, bleeding, mini-PCNL

## Abstract

**Background:**

Fused renal pyramid (FRP) is a kidney anatomical structure which was first identified by us. The vascular anatomy of FRP exhibits different from that of the normal renal pyramid (NRP), manifested by the distribution of the ectopic interlobar arteries in FRP. In this study, we analyzed the effect of FRPs on bleeding when using calyx access in mini-percutaneous nephrolithotomy (PCNL).

**Patients and Methods:**

Overall, 633 patients who underwent ultrasound-guided single-tract mini-PCNL were divided into two groups according to the puncture method used: in group A, puncture was performed through the axial direction of the renal calyx, the line from the apex of the fornix to the center of the neck plane under B-mode ultrasound guidance; and in group B, Doppler ultrasound-guided axillary puncture through calyces corresponding to NRPs when the plane of renal column blood vessels on both sides was selected or calyx puncture through the hypovascular area of the FRPs. Relevant demographic and clinical data were retrospectively analyzed.

**Results:**

The two groups exhibited similar baseline characteristics. No significant differences were found in hemoglobin reduction, puncture site, tract size, postoperative creatinine level, or stone-free rate between the two groups (*P* > 0.05). Blood transfusion and embolization rates in group B were significantly lower than those in group A (*P* = 0.03 and 0.045, respectively). No differences were found between the two groups in terms of persistent pain, hydrothorax, fever, subcapsular hematoma, and urosepsis (*P* > 0.05). The overall complication rate was not significantly different between the two groups (*P* = 0.505).

**Conclusions:**

FRP is a non-negligible anatomical structure that may cause hemorrhage when using calyx access. Doppler ultrasound can identify ectopic blood vessels in FRPs to reduce bleeding during calyx access in mini-PCNL procedures.

## Introduction

Percutaneous nephrolithotomy (PCNL) is a minimally invasive modality that is widely used for the management of upper urinary tract stones and is recommended as the first-line treatment for staghorn stones in most patients (1, 2). Although mini-PCNL, which is defined as a tract size between 14 and 22 Fr, has become mainstream and the development of access miniaturization in the past two decades has significantly reduced surgical injury (3), bleeding remains one of the most common complications of PCNL, with some contemporary series reporting a blood transfusion rate of 3%–6% (4–6). PCNL-related renal hemorrhage can be caused by various factors, with vascular injury to the renal parenchyma being the most direct cause (7).

The relationship between the collecting system and vascular anatomy is the basis for establishing safe access, and choosing an appropriate puncture pathway can help reduce bleeding (8, 9). Currently, the renal calyx serves as a puncture landmark irrespective of whether ultrasound or fluoroscopy is used for PCNL access (10, 11). The neck-fornix axis line of the calyx is considered a safe puncture path because, theoretically, it coincides with the centerline of the renal pyramid, which is the minimum injury line to the renal interlobar artery during puncture according to an anatomical study of cadaveric kidneys by Sampiao et al. (11). However, although puncture at the axis line of the calyx has been widely used, severe bleeding occurs occasionally (8, 12).

**Figure 1:**
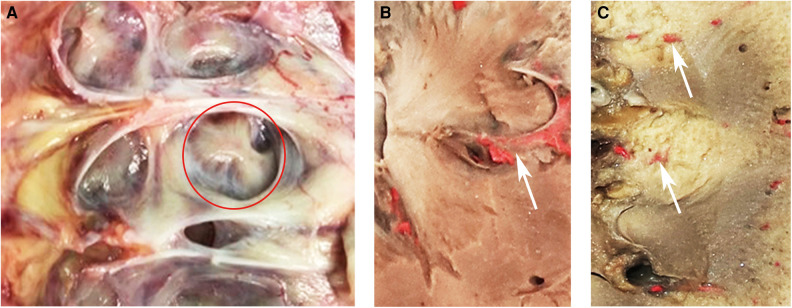
(**A**) FRP in a nephrectomy specimen. There are two papillae entering a compound calyx. The red circle indicates the FRP. (**B**) FRP in a human kidney. There are two renal pyramids fused into one FRP. The white arrow indicates the interlobar blood vessel distributed in the FRP. (**C**) NRP in a human kidney. The white arrow indicates the interlobar artery distributed in the renal column. FRP, fused renal pyramid; NRP, normal renal pyramid.

The renal pyramid is generally considered to be a relatively hypovascular area. There are two different types of renal pyramids: the normal renal pyramid (NRP) and the fused renal pyramid (FRP). The NRP exhibits a one-to-one anatomical relationship with the simple minor renal calyx, whereas the FRP refers to two or more adjacent pyramids fused in the renal medulla, which enter the compound minor renal calyx together. The FRP is an anatomical structure of the kidney that we first identified, with a vascular anatomy distinct from that of the NRP due to the distribution of the ectopic interlobar artery inside it (Figures 1A–C). Additionally, in a previous study, we observed that by simulating percutaneous access in a porcine kidney, vascular damage caused by puncture of the centerline of the FRP was more significant at the pathological level, and puncturing from the side pyramid of the FRP could effectively avoid the ectopic interlobar artery in the FRP (13).

The ultrasound-guided puncture procedure in our research center uses a calyx access. The calyx corresponding to the FRP is a well-described concept of the compound calyx. FRP challenges the existing method of puncture using the calyx axis, and it remains to be determined whether axial puncture is equally safe for the compound calyx. Our hypothesis is that the abnormal vascular anatomy of the FRP may have an impact on the choice of puncture pathway when using the calyx access in mini-PCNL. In this study, we used color Doppler ultrasonography to identify vessels in FRP to adjust the puncture pathway in mini-PCNL procedures and compared the procedure with previous puncture methods to assess the possible effect of FRP on calyx puncture and its clinical value.

## Patients and Methods

### Mini-PCNL Using Different Puncture Methods Under Ultrasound Guidance

We retrospectively reviewed an institutional review board-approved clinical database of endourological procedures and identified consecutive patients diagnosed with kidney stones or upper ureteral stones who underwent ultrasound-guided mini-PCNL in the prone position over a 3-year period (January 2018 to June 2021). Individuals aged <18 years, those who underwent second-stage PCNL or surgery through preoperative indwelling accesses, and those with multiple tracts, anatomical kidney abnormalities (such as horseshoe kidney and ectopic kidney), and coagulation dysfunction were excluded. This study was reviewed and approved by the Ethics Review Committee of the Clinical Study of Renmin Hospital of Wuhan University, and written informed consent was waived by the IRB (approval no. WDRM2019-K030).

**Figure 2:**
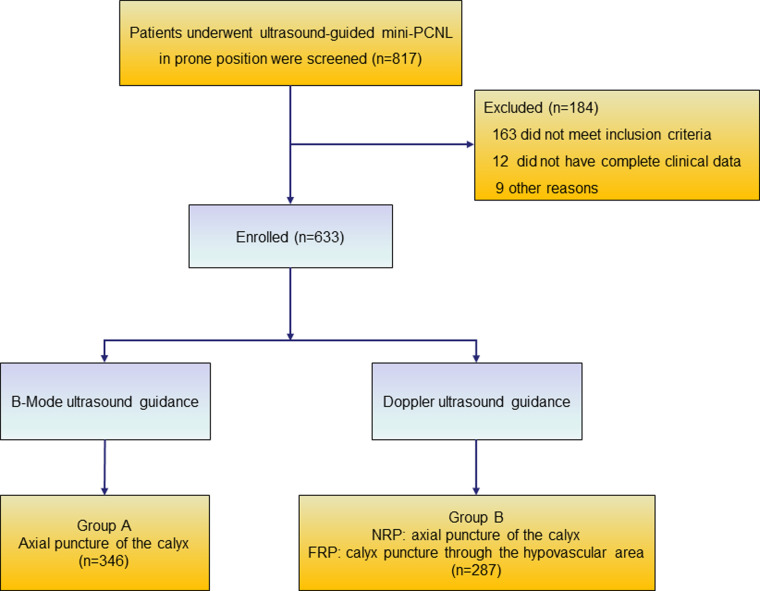
Flow diagram of excluded and grouped patients. NRP, normal renal pyramid; FRP, fused renal pyramid.

**Figure 3:**
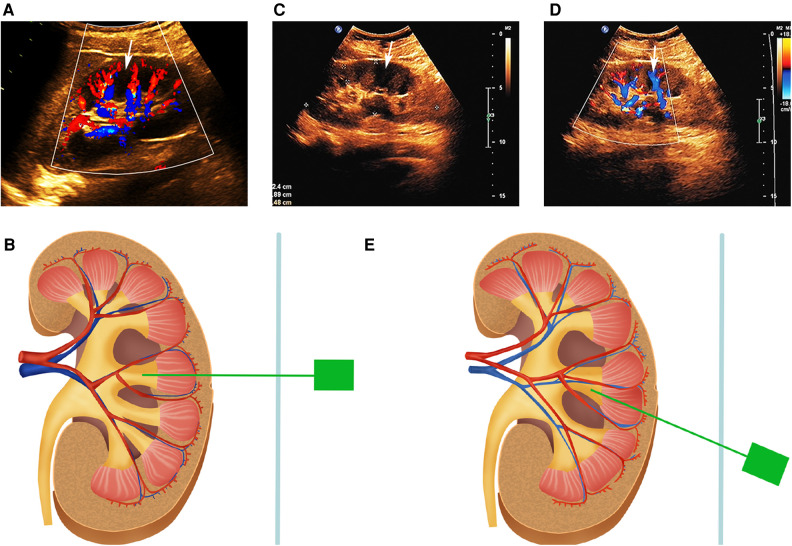
(**A**) NRP and the simple calyx in Doppler ultrasound. There are blood flow signals in the renal columns on either side of the NRP. The white arrow indicates the NRP. (**B**) Schematic representation of the axial puncture of the calyx corresponding to NRP. (**C,D**) FRP and the compound calyx in B-mode ultrasound and Doppler ultrasound. The white arrow indicates the FRP. Color flow signals exist within FRP. (**E**) Schematic representation of calyx puncture through the hypovascular area of the FRP. NRP, normal renal pyramid; FRP, fused renal pyramid.

A total of 633 patients who met the inclusion criteria were included in this study and divided into two groups according to the different puncture methods used in different periods (Figure 2). Case data that fulfilled the research requirements were divided into two groups based on the different intraoperative puncture methods used during different periods. From January 2018 to June 2019, puncture was performed through the axial direction of the renal calyx, the line from the apex of the fornix to the center of the neck plane, under B-mode ultrasound guidance (Supplementary Figure S1) (group A). Following the discovery and characterization of the FRP structure, the puncture method was adjusted accordingly under ultrasound guidance. From July 2019 to June 2021, a calyx puncture was performed under Doppler ultrasound guidance to avoid the blood vessels in the pyramid (group B). First, the target calyx was identified on B-mode ultrasound, and then the probe was rotated to locate the plane of coincidence between the target calyx and vessels of the renal columns on both sides. When there was no obvious blood vessel distribution in the pyramid corresponding to the punctured calyx, we rotated the ultrasound probe to ensure that the target calyces and both renal column vessels were in the same plane, and the plane was fixed and punctured in the axial direction of the target calyx (Figures 3A,B). If there was a significant blood flow in the puncture path, the probe was moved laterally until there was no significant blood flow in the puncture line. Subsequently, puncture was performed through the hypovascular area on the side of the FRP to avoid blood vessels (Figures 3C–E). Ultimately, we performed a total of 61 (21.3%) calyx punctures through the hypovascular area of the FRPs on patients in group B.

All patients underwent standard preoperative evaluation, including history, physical examination, computed tomography (CT) urography, and laboratory investigations, including urine culture. Some patients with renal insufficiency are diagnosed using non-contrast CT and renal radionuclide dynamic imaging. Patients with tract infections were treated routinely with culture-appropriate antibiotics up to the day of the procedure.

### Surgical Technique

After the induction of general anesthesia, the patient was placed in the lithotomy position, and a 5–7-F ureteric catheter was inserted retrogradely into the affected side. The patient was then repositioned to the prone position. According to the distribution of stones and the morphology of the collection system in preoperative CT images, ultrasound was used to determine the target renal calyces. In patients with multiple stones, the choice of the target calyx was aimed at creating access that could reach all calyces that contained stones.

An 18-gauge coaxial needle was inserted into the skin under real-time ultrasound guidance using a freehand technique. The direction of the puncture needle was adjusted before the tip penetrated the kidney parenchyma. After determining kidney movement with respiration, the needle was advanced to accurately puncture the target calyx according to a predetermined path. In actual operations, adverse effects caused by the position of the ribs and the direction of the renal calyx are often observed. Generally, a small deviation from the direction of the predetermined path does not negatively affect the overall procedure; however, the puncture should be as close as possible to the predetermined path.

Successful placement was assumed when there was free flow of irrigation fluid from the needle hub after removal of the stylet. A 0.089-cm flexible-tip U-shaped guidewire was inserted through the needle sheath into the collecting system. Tract dilatation was performed serially using fascial dilators with appropriate access sizes. A Karl Storz nephroscope was used for mini-PCNL. Holmium lasers were used for stone fragmentation. A double-J tube and nephrostomy tube were inserted per routine postoperatively. In this study, surgery was performed by only four experienced surgeons (F.C., WM.Y., T.R., and Y.R.), who are skilled in ultrasound-guided mini-PCNL. Each had performed >300 mini-PCNL procedures during their careers (14, 15).

### Postoperative Evaluation

A decrease in hemoglobin (Hb) level was determined by comparing the preoperative HB level with that at 24 h postoperatively. If intraoperative bleeding occurred, the Hb level at the emergency postoperative checkup prevailed. An Hb level ≤80 g/L is the critical value for postoperative whole blood transfusion therapy. Before discharge or second-stage surgery, patients underwent kidney-ureter-bladder radiography or non-contrast CT to assess the stone-free rate (SFR), defined as no residual fragments ≥0.4 cm (16). Shockwave lithotripsy, flexible ureteroscopy, and second-stage mini-PCNL are auxiliary procedures for managing significant residual stones (17). Postoperative complications were classified according to the modified Clavien-Dindo classification (18).

### Statistical Analysis

Statistical analysis was performed using Statistical Package for the Social Sciences version 25.0. Continuous variables are presented as mean ± standard deviation and analyzed using one-way analysis of variance and Student’s *t*-test. Enumeration variables, presented as numbers and percentages, were analyzed using the chi-squared test and Fisher’s exact test. Differences were considered statistically significant at *P* < 0.05.

## Results

The baseline characteristics of the patients and their operative and postoperative characteristics are summarized in Table 1. The stone size was significantly larger in group B than in group A (*P* = 0.037); all other patients’ demographic variables and preoperative clinical characteristics were comparable between the two groups.

**Table 1:** Patients’ demographic, operative and postoperative clinical characteristics.

Characteristics	Group A	Group B	*P*
Number of patients	346	287	
Age, years, mean (SD)	55.6 (12.0)	54.6 (11.7)	0.292
Gender-male/female, *n*	217/129	174/113	0.622
Stone size-maximal diameter, cm	2.07 (0.99)	2.24 (1.05)	0.037
Laterality–left/right, *n*	178/168	151/136	0.811
Stone type, *n* (%)			0.226
Single	116 (33.5%)	96 (33.4%)	
Multiple	199 (57.5%)	158 (55.1%)	
Staghorn	31 (9.0%)	33 (11.5%)	
Stone location, *n* (%)			0.293
Calyx	159 (46.0%)	126 (43.9%)	
Pelvis	116 (33.5%)	107 (37.3%)	
Upper ureter	71 (20.5%)	54 (18.8%)	
Grade of hydronephrosis, *n* (%)			0.246
None or mild	183 (52.9%)	146 (50.9%)	
Moderate	71 (20.5%)	77 (26.8%)	
Severe	92 (26.6%)	64 (22.3%)	
Comorbidities, *n* (%)			
Hypertension	110 (31.8%)	113 (39.4%)	0.186
Diabetes mellitus	33 (9.5%)	38 (13.2%)	0.164
Hyperuricemia	20 (5.8%)	24 (8.4%)	0.213
Previous stone surgery	108 (31.2%)	107 (37.3%)	0.110
Preoperative serum creatinine level, µmol/L, mean (SD)	82.8 (40.2)	82.3 (40.5)	0.787
Puncture sites, *n* (%)			0.285
Upper calyx	18 (5.2%)	14 (4.9%)	
Middle calyx	277 (80.1%)	234 (81.5%)	
Lower calyx	51 (14.7%)	39 (13.6%)	
Tract size, *n* (%)			0.236
16Fr	54 (15.6%)	60 (20.9%)	
18Fr	2 (0.6%)	3 (1.0%)	
20Fr	255 (73.7%)	206 (71.8%)	
22Fr	35 (10.1%)	18 (6.3%)	
Postoperative serum creatinine level, µmol/L, mean (SD)	84.6 (42.5)	83.4 (41.2)	0.472
Hb drop, g/L, mean (SD)	12.68 (9.80)	12.07 (9.01)	0.527
Stone free after one stage operation, *n* (%)	248 (71.7%)	213 (74.2%)	0.530

*Hb, hemoglobin; SD, standard deviation.*

We compared the operative and postoperative characteristics of groups A and B. The decrease in Hb level in group B was lower than that in group A. However, there was no statistically significant difference between the two groups in terms of the mean postoperative Hb level (*P* = 0.527). No significant differences were found in puncture site, tract size, postoperative creatinine level, or SFR between the two groups (*P* > 0.05).

Clavien–Dindo grade complications are summarized in Table 2. Blood transfusion and angioembolization rates were significantly lower in group B than in group A (*P* = 0.03 and 0.045, respectively). No differences were found between the two groups in terms of persistent pain, hydrothorax, fever, subcapsular hematoma, and urosepsis (*P* > 0.05). None of the patients in group B experienced septic shock. The overall complication rate was not significantly different between the two groups (*P* = 0.505).

**Table 2:** Comparison of postoperative complications stratified according to puncture method, *N (%).*

Complications	Clavien–Dindo grade	Group A	Group B	*P*
Persistent pain	I	7 (2.0%)	8 (2.8%)	0.604
Hydrothorax	I	6 (1.7%)	7 (2.4%)	0.582
Subcapsular haematoma	II	17 (4.9%)	13 (4.5%)	0.853
Blood transfusion	II	18 (5.2%)	3 (1.0%)	0.003
Angioembolisation	IIIb	8 (2.3%)	1 (0.35%)	0.045
Fever (>38.5°C)	I	20 (5.8%)	14 (4.9%)	0.724
Urosepsis	IVa	9 (2.6%)	11 (3.8%)	0.495
Septic shock	IVb	5 (1.4%)	0	/
Overall complications	I–II	35 (10.1%)	27 (9.4%)	0.790
	IIIb–IVb	20 (5.8%)	13 (4.5%)	0.591

## Discussion

With advances in technology and equipment, PCNL-related complications have reduced significantly; nevertheless, severe bleeding occurs occasionally, and considerably affects surgical efficacy and patients’ prognosis (7). Severe PCNL-related renal hemorrhage is usually associated with injury to the segmental and interlobar arteries in the renal parenchyma, and venous bleeding is easily controlled using conservative measures (19).

Calyx puncture itself is beneficial for reducing injury to the blood vessels in the renal column. In PCNL, the anatomical basis of the renal artery and collection system is used to select the center of the renal pyramid rather than the renal column or infundibulum as the puncture site, because the renal pyramid does not contain blood vessels traveling through it. Therefore, B-mode ultrasound-guided puncture is feasible because no vascular damage needs to be considered when puncturing the renal pyramid. After our extensive study of the FRP, we realized that the traditional anatomical experience needed to be updated and that the presence of blood vessels traveling within fused pyramids was key to its differentiation from NRPs, and that color Doppler will play a role in detecting blood vessels. Because there is little to no vascularity in normal pyramids, if color Doppler detects blood flow, the renal pyramid must be the FRP. In group B, axis puncture of the normal calyx and hypovascular area puncture of the compound calyx were performed to exploit the advantages of Doppler to avoid ectopic blood vessels in the pyramid. Additionally, although the stone was larger in group B, the incidence of bleeding-related complications was significantly decreased, which may be related to technological advances including puncture methods.

Based on the safety principles for renal vascular anatomy, percutaneous renal puncture is usually performed in less vascular areas (20). For NRP, a puncture through the axis line of the simple calyx under ultrasound guidance can reduce vascular injury and bleeding; however, the opposite is true for FRP. Taking the axis line of the compound calyx as a puncture path, it may pass through the vascular variation area inside the FRP. Although puncture does not necessarily directly damage the interlobular vessels within the FRP, tract dilation and movement of the mirror sheath can increase the risk of bleeding, as the FRP itself is a vascular distribution area. This may be an anatomical cause of overt bleeding due to a seemingly precise calyx puncture. Although fusion of three or more pyramids rarely occurs, especially in the middle group calyces (13), the use of Doppler ultrasound to adjust the puncture path to avoid ectopic blood vessels can reduce bleeding.

The contrast of the renal pyramid is not obvious on ultrasound, and an FRP, in the anatomical sense, is difficult to identify using B-mode ultrasound alone. Doppler ultrasound is more sensitive to blood vessels and can fully depict the distribution of kidney arteries, such as the renal segmental, interlobar, and cortical arteries (21), and is an effective imaging modality that can identify the blood vessels distributed in the renal column and FRP (Supplementary Figure S2). Fluoroscopy can clearly depict the shape of the intact collecting system, which is highly effective for puncturing the non-dilated calyx and improving the puncture success rate (22). However, it is difficult to identify ectopic blood vessels using fluoroscopy alone. Therefore, the positive results of this study are difficult to replicate by using fluoroscopy-guided puncture alone. Contrast-based CT clearly depicted the FRP in the cortical phase and fully displayed the morphology of the renal calyx in the secretory phase (Supplementary Figure S3), which is clinically significant for planning the puncture path before surgery.

Currently, some researchers believe that non-papillary puncture is as safe as papillary puncture, illustrating that the bleeding rate of the two puncture pathways did not differ statistically, and that the bleeding rate of non-papillary puncture was close to that reported in the literature for papillary puncture (23–25). Based on our preliminary autopsy of cadavers, we found that the prevalence of FRP in human kidneys is not low (13). This indicates that some papillary punctures actually involve puncture of the FRP and explains why bleeding still occurs according to calyx axis puncture. Furthermore, papillary puncture may not always be equivalent to hypovascular puncture, and this adds to the uncertainty of papillary puncture bleeding. The bleeding rate of papillary puncture may be amplified in the absence of knowledge of the presence of FRPs. In other words, in the presence of FRP, papillary punctures face a very similar vascular anatomic environment to that of non-papillary punctures. Our study is important because it refines the principles of calyx access, as the current principles of papillary puncture are imperfect and do not consider the structural variations that are common in the human kidney.

We propose the concept of FRP for the first time. FRP is a new anatomical variation that increases the risk of bleeding when using calyx access in PCNL, suggesting that calyx puncture needs to be more refined and timelier adjusted according to the vascular anatomy of the renal pyramids. Furthermore, this study is the first to demonstrate that identifying blood vessels within the FRP using color Doppler ultrasound and designing a safe puncture pathway can reduce the occurrence of bleeding, which may help urologists to further reduce bleeding complications associated with mini-PCNL in the future. Overall, this study extends the role of kidney-related anatomy in PCNL by providing new insights into reducing calyx access-related bleeding.

The present study has several limitations. First, this was a retrospective, single-center study. Conducting prospective studies will obtain more comprehensive evidence to support our conclusions, which is in our plan. Second, although we were proficient in the related operations of ultrasound-guided calyx puncture before starting this study, advances in technology and equipment may have had an impact on our results. Third, although our study center can accurately identify ectopic vessels in FRP using color Doppler ultrasound, other study centers may not be able to replicate this result without a specific focus on FRP. It is important to note that although the objective fact that FRP may increase the risk of vascular injury during calyx access cannot be ignored, the presence of this risk does not necessarily mean that bleeding must occur. The use of color Doppler ultrasonography to identify the plane of lack of vessels that can avoid vessels within the FRP for puncture is an effective strategy that can minimize bleeding due to the application of the renal calyx pathway.

## Conclusions

Although the safety of calyx axis puncture is more widely recognized, FRP cannot be ignored as new anatomical evidence that may cause bleeding when using calyx access, indicating that the establishment of percutaneous access requires a more refined approach to the vasculature and specific anatomy of the kidney. Doppler ultrasound may be a reliable tool for puncture guidance that can identify ectopic blood vessels in the FRP and help adjust the puncture path in time to reduce bleeding when performing calyx access in mini-PCNL procedures.

## Data Availability

The original contributions presented in the study are included in the article/Supplementary Material, further inquiries can be directed to the corresponding author/s.
