# Supplementary figures and images for: Presence of a Novel Anatomical Structure May Cause Bleeding When Using the Calyx Access in Mini-Percutaneous Nephrolithotomy
Source: Front Surg. 2022 Jun 21;9:942147. doi: 10.3389/fsurg.2022.942147 (PMC9253458; doi:10.3389/fsurg.2022.942147)

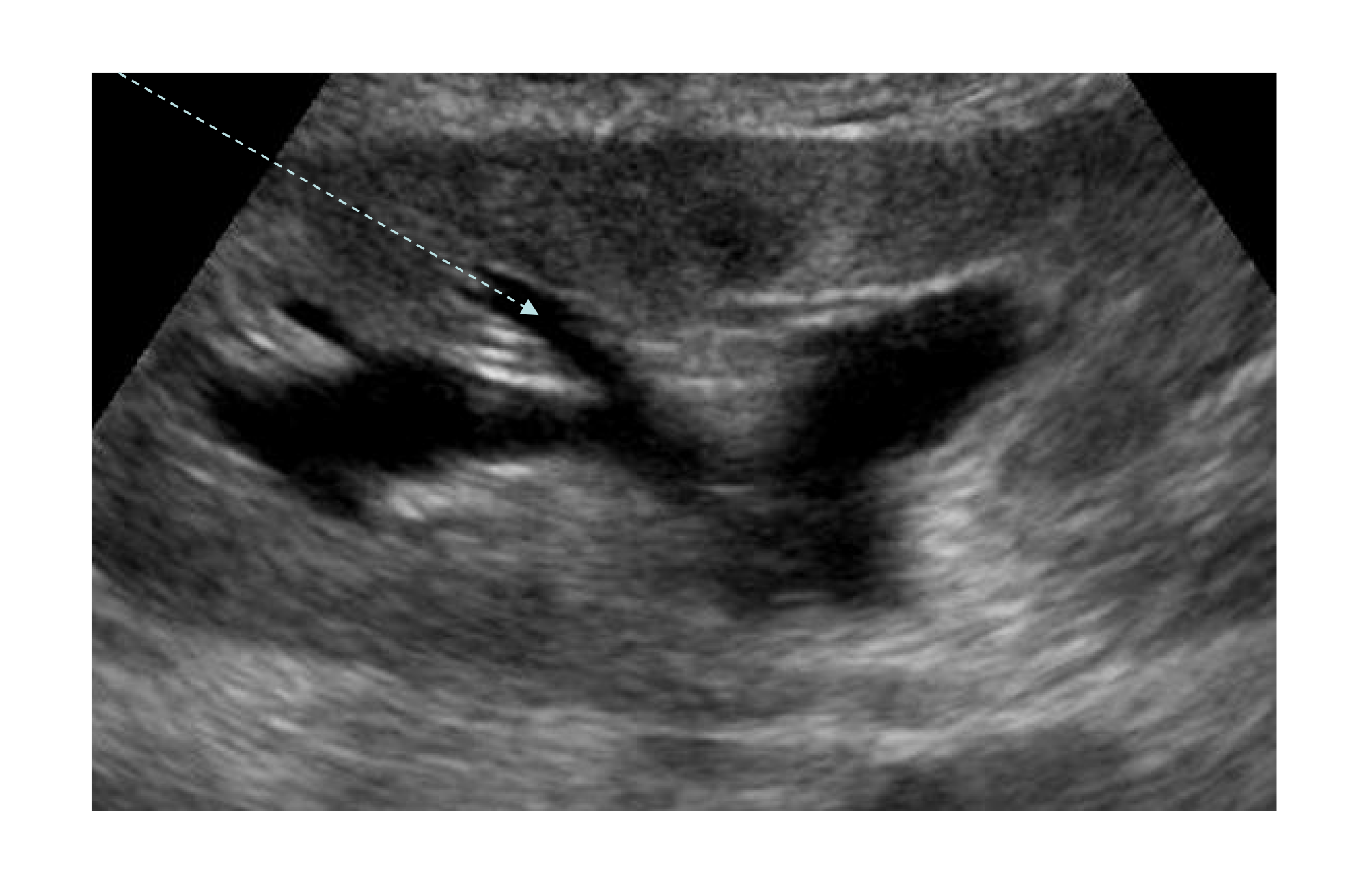

Supplement: Supplementary file 1 [file Image_1_v1.tif]

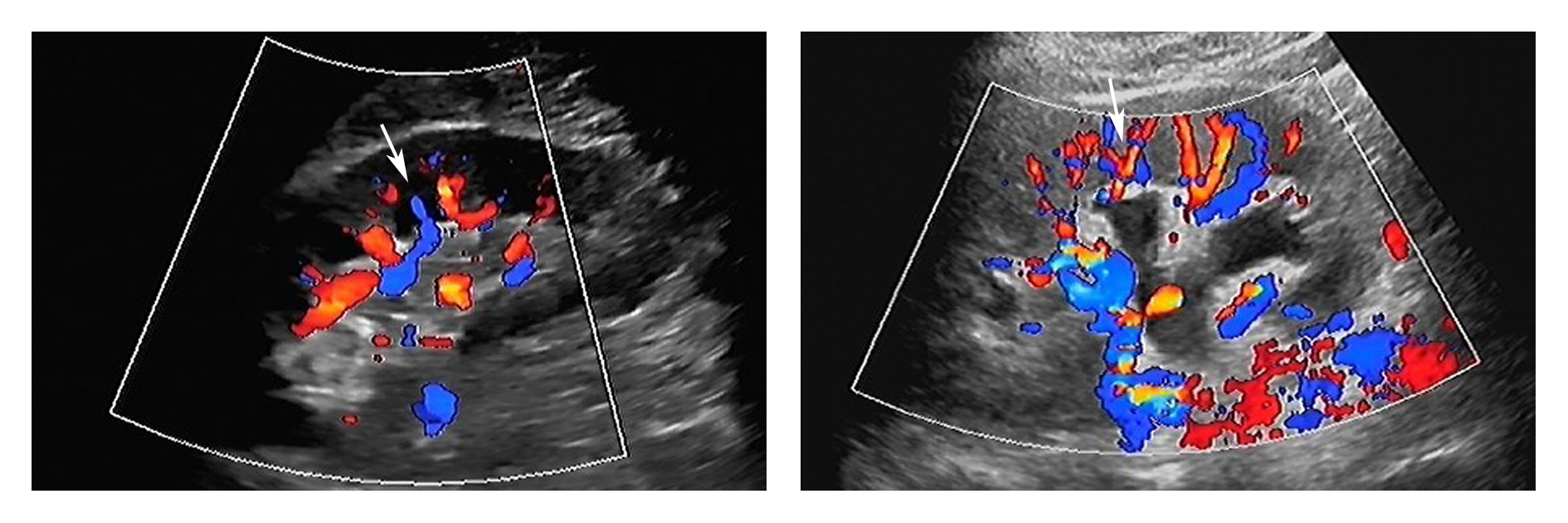

Supplement: Supplementary file 2 [file Image_2_v1.tif]

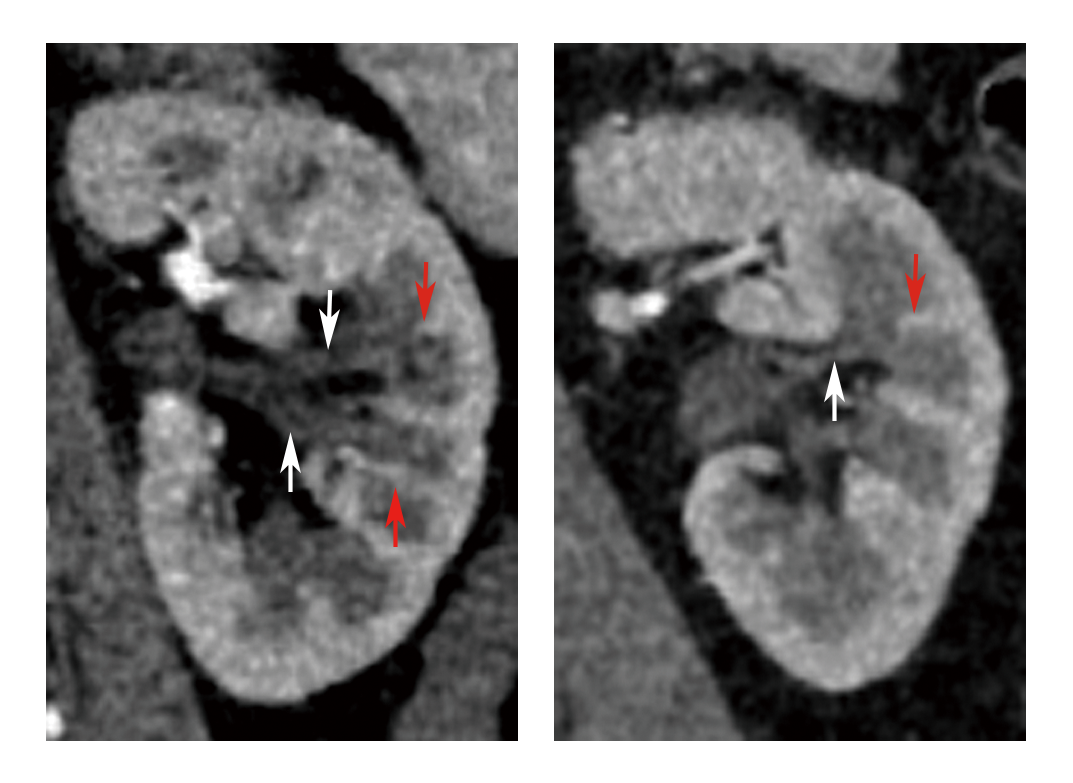

Supplement: Supplementary file 3 [file Image_3_v1.tif]
